# Evidence accumulation from experience and observation in the cingulate cortex

**DOI:** 10.1038/s41586-025-09885-0

**Published:** 2026-01-07

**Authors:** Ruidong Chen, Setayesh Radkani, Neelima Valluru, Seng Bum Michael Yoo, Mehrdad Jazayeri

**Affiliations:** 1 Department of Brain & Cognitive Sciences, Massachusetts Institute of Technology, Cambridge, USA; 2 McGovern Institute for Brain Research, Massachusetts Institute of Technology, Cambridge, USA; 3 Department of Biomedical Engineering, Sungkyunkwan University, Suwon, Republic of Korea; 4 Howard Hughes Medical Institute, Massachusetts Institute of Technology, Cambridge, USA

## Abstract

We use our experiences to form and update beliefs about the hidden states of the world^1–3^. When possible, we also gather evidence by observing others. However, how the brain integrates experiential and observational evidence is not understood. We studied the dynamics of evidence integration in a two-player game with volatile hidden states. Both humans and monkeys successfully updated their beliefs while playing the game and observing their partner, though less effectively when observing. Electrophysiological recordings in animals revealed that the anterior cingulate cortex integrates independent sources of experiential and observational evidence into a coherent neural representation of dynamic belief about the environment’s state. The geometry of population activity revealed the computational architecture of this integration and provided a neural account of the behavioral asymmetry between experiential and observational evidence accumulation. This work lays the groundwork for understanding the neural mechanisms underlying evidence accumulation in social contexts within the primate brain.

A hallmark of cognition is the ability to infer the hidden causes of our experiences. Waking up with an upset stomach, you might wonder if it is due to food poisoning or if you caught the flu. Your first clues are your symptoms—nausea versus fever would hint at different causes. But you may also rely on others’ experiences. You may think it is flu if a coworker recently had the flu, or you may reason it is food poisoning if your dinner partner has similar symptoms. While the capacity to integrate experiential and observational evidence to infer hidden causes is unequivocal, the neural mechanisms that enable such sophisticated computations are not well understood.

The anterior cingulate cortex (ACC) is thought to play a central role in evidence-based decision making. ACC carries signals related to outcome history, performance monitoring, action and strategy selection, and beliefs about associations and contexts^1–21^. Notably, ACC representations persist over relatively long time scales^22^ and integrate information across events and experiences^1–3,23^. These findings coupled with complementary causal studies^3,15,24–26^ have provided strong evidence that ACC encodes behaviorally-relevant beliefs about latent causes in the environment.

We know far less about the computational and neural basis of observational inference and learning. Some studies have examined the neural signatures of observed reward and punishment^27^ in the amygdala, striatum, and many cortical areas^28–39^. Among these, ACC has been a prominent region of interest that supports vicarious reinforcement and observational fear conditioning^33,35,36,39–42^. Therefore, ACC may play a more general role in belief updating that spans both experiential and observational settings.

However, most studies on observational learning have relied on relatively simple tasks that either do not require inferring latent causes or do not involve integration of experiential and observational evidence. As a result, little is known about the parallels and distinctions between the neural representation of experiential and observational evidence, and how these two sources of information are integrated to form beliefs about the latent state of the world.

Here, we tackle these questions using a combination of human behavior, primate neurophysiology, and neural modeling in a two-player belief-updating game. The behavioral results revealed a familiar asymmetry between experiential and observational evidence^43–45^ in both humans and monkeys. ACC recordings revealed how the evidence derived from self and other experiences are integrated into a coherent population pattern of neural activity supporting participants’ beliefs and behavior on a trial-by-trial basis. Moreover, the organization of the population activity associated with self-experience, observation, and integrated belief provided an explanation for the behavioral asymmetry.

## Behavioral task and performance

**Figure 1.**
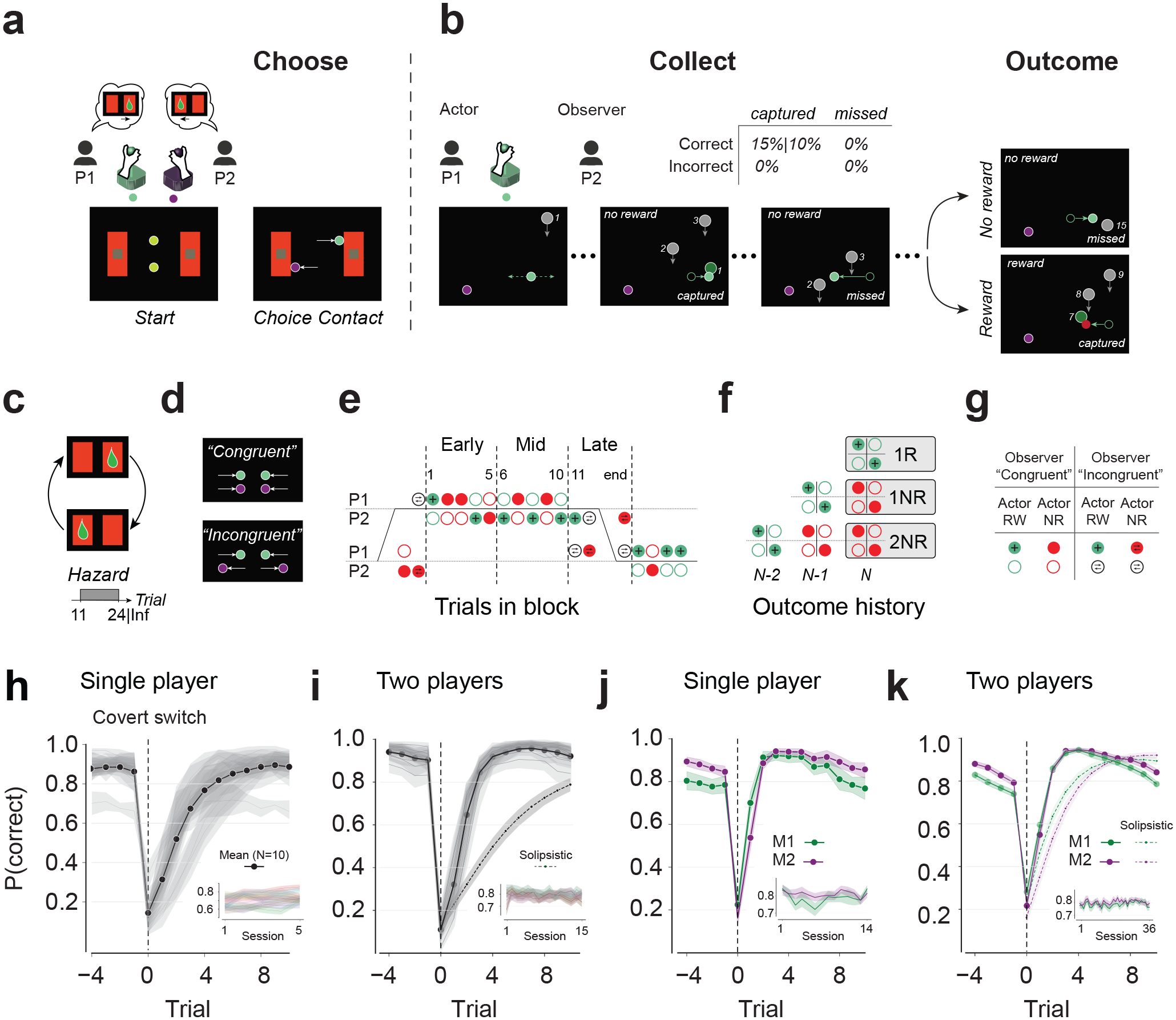
Multi-agent evidence integration task and performance (**a**-**b**) Trial structure. (**a**) Phase 1. Players choose one of two arenas (“Choose”). Start: two avatars are presented in the middle of two arenas. Choice contact: When an avatar contacts an arena, the choice is registered and the avatar stops moving. (**b**) Phase 2. The Actor plays a token collection game (“Collect”) for reward (“Outcome”). The Observer appears below their chosen arena. The trial ends after reward or 15 unrewarded tokens. Outcome: The Actor receives reward probabilistically for correct choices (table above). (**c**) Covert switch schedule. On each trial, only one arena has a non-zero reward probability (green drop). (**d**) In phase 1, players can choose the same (“Congruent”) or different arenas (“Incongruent”). (**e**) Example run. Gray lines denote arenas. Correct arena switches in blocks (piecewise black line). Disks show trial conditions (Actor/Observer: filled/open; left/right: near top/bottom horizontal line; rewarded/unrewarded: green/red; incongruent Observer: opposite arrows). Numbers indicate trial in block starting at the first rewarded trial (i.e., subjective block switch). Trials 1–5, 6–10, and beyond 11, demarcated by dashed lines, are Early, Mid, and Late subdivisions within a block. (**f**) Outcome history nomenclature. 1R: congruent rewarded trial. 1NR, 2NR: successive congruent unrewarded trials in the same arena following 1R. (**g**) Key for Actor/Observer and reward conditions in **e**-**f**. (**h**) Single-player human performance. Proportion correct, P(correct), aligned to covert block switches (trial 0). Black line = mean across participants; Shade = 95% confidence interval. Inset: P(correct) over sessions, each line corresponds to one participant. (**i**) Two-player human performance. Solid: mean performance of all participants. Dashed: solipsistic model. Human performance was significantly better than the solipsistic model (p=2.9e-28, two sided t-test). (**j**-**k**) Same as **h**-**i** for the two monkeys. Two-player performance was significantly better than the solipsistic model (p=4.4e-8 for M1, p=6.7e-19 for M2, two sided t-test).

We designed a two-player game for humans and monkeys to investigate the behavioral and neural signatures of updating beliefs in the presence of both experiential and observational evidence (Figure 1a–g). Each trial consists of two phases. In the first phase, the players use their respective joysticks to independently choose between a left and a right arena (Figure 1a). They are free to choose either arena (Figure 1d) but this choice is consequential because only one of the arenas may eventually lead to a reward (Figure 1c). Human participants additionally reported their choice confidence (see Methods).

In the second phase, one player is randomly designated as the actor. The actor controls an avatar in their chosen arena with the joystick and must capture tokens falling from the top of the screen, aiming to collect as many as possible to maximize expected reward. Receiving reward is probabilistic and depends on both the arena and the number of captured tokens during the second phase (Figure 1b). If the actor selects the correct arena, each captured token has a fixed probability of yielding a reward (0.1 for humans and 0.15 for monkeys), and the delivery of reward would terminate the trial (i.e., no more token capture allowed). In the other arena, capturing tokens never results in a reward. Trials without rewards end after all 15 tokens have dropped. The correct arena switches in a blocked fashion (Figure 1c). From the moment the actor begins to collect tokens to the end of the trial, the other player, whom we refer to as the observer, watches the actor play and witnesses the outcome without receiving any reward.

We matched the actor’s and observer’s sensory experiences as closely as possible: both saw all events, the actor was designated only after both chose an arena, and roles were assigned randomly. Thus, both had equal information for rational inference, ensuring any asymmetries in evidence accumulation reflect internal rather than external factors.

We collected data from ten humans (five pairs) and two monkeys. All participants learned the task in a single-player version (average choice performance (mean±SD) in humans: 71.85±7.02% across 5 sessions, Figure 1h; in M1: 78.39±3.34% and in M2: 80.96±1.65% across 14 sessions, Figure 1j) before moving onto the two-player version (performance in humans 79.15±3.05% over 15 sessions, Figure 1i; in M1 78.68±2.07%, and M2 80.38±1.73% over 36 sessions, Figure 1k). Monkeys trained longer and faced higher reward probabilities. In both versions, performance dropped immediately after covert block switches and recovered within a few trials, indicating belief updating (Figure 1h–k, S2c–f). We used single-player data to simulate “solipsistic” agents that ignored observer trials (see Methods). As expected, solipsistic agents performed worse than their respective single-player source due to ignored observer trials (humans: 50.72±10.90%; M1: 74.82±3.27%; M2: 71.40±3.09%), and significantly worse compared to the two-player sessions (Figure 1i,k). Attention to observer trials was evident in monkeys’ eye movement data, with preferential orienting of gaze toward the active arena (M1: 58.0 ± 22.5%; M2: 87.4 ± 18.7%; Figure S1h,k).

## Humans and monkeys integrate experiential and observational evidence rationally

**Figure 2.**
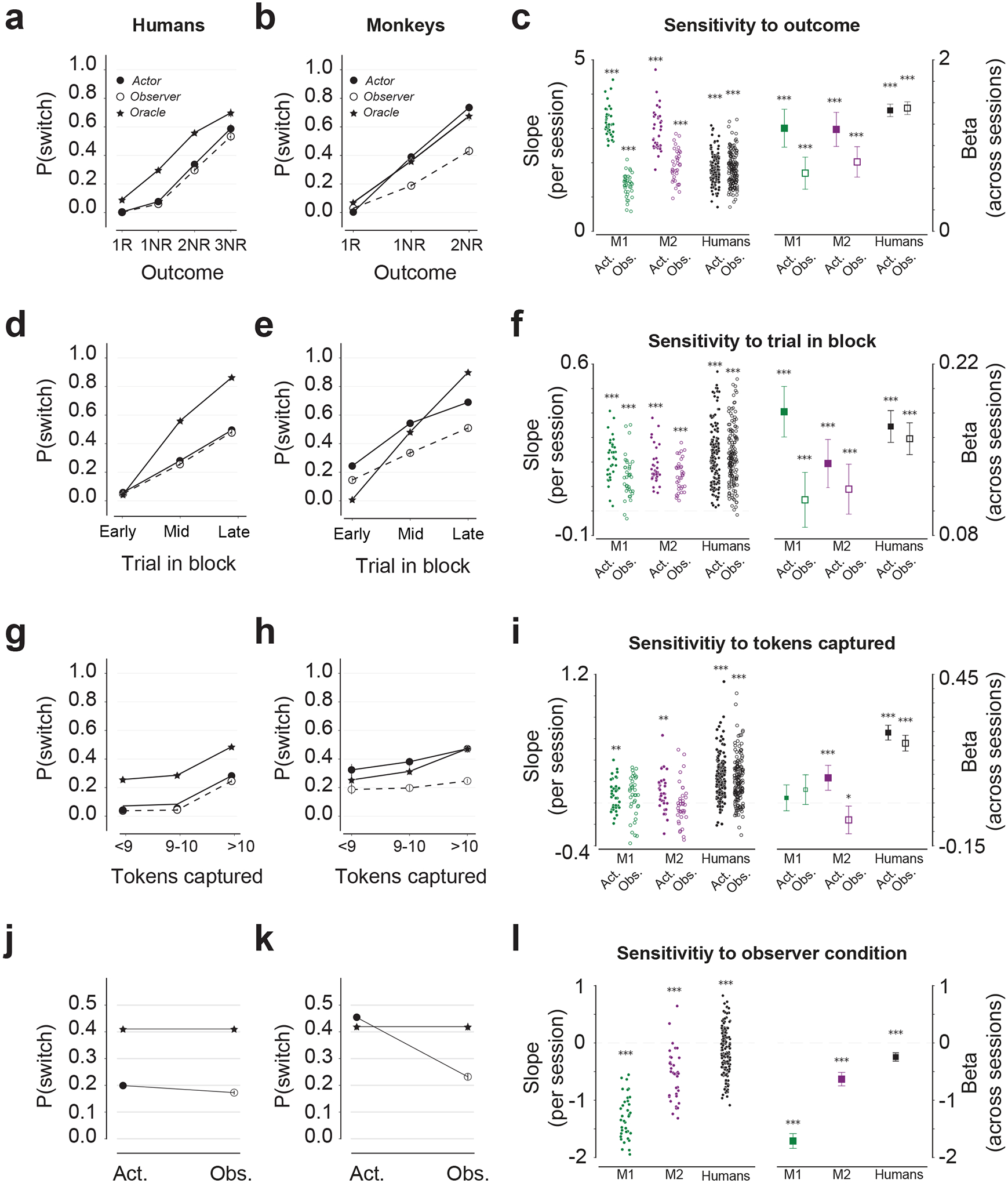
Behavioral characteristics of experiential and observational learning in monkeys and humans (**a**-**b**) Switch probability, P(switch) as a function of trial outcome for congruent trials across humans (**a**) and monkeys (**b**). Results are shown separately for Actor (filled circle), Observer (open circle), and Oracle (star). 1R: rewarded trials. *n*NR: *n-th* consecutive unrewarded trial within the same arena. (**c**) Left: Regression slope relating P(switch) to outcome history, separately for Actor (filled) and Observer (open) for each participant (two colors) for each session. N=36 sessions for M1 and M2, 75 sessions for humans (5 pairs of 15 sessions each). Stars indicate statistical significance (two-sided t test; ***:P<0.001, **:P<0.01, *:P<0.05). Right: beta values of a multivariable logistic regression relating P(switch) to outcome history, trial in block, and number of captured tokens for Actor (filled) and Observer (open) trials for each participant across sessions. Data are presented as mean values +/− 95% confidence interval; stars indicate statistical significance (two-sided t test; ***:P<0.001, **:P<0.01, *:P<0.05). (**d**-**f**) Same as (**a**-**c**) for the relationship between P(switch) and trial position in the block for congruent unrewarded trials. (**g**-**i**) Same as (**a**-**c**) for the relationship between P(switch) and the number of tokens captured for congruent unrewarded trials. (**j**-**l**) Same as (**a**-**c**) for the relationship between P(switch) and player identity (Actor versus Observer) for congruent unrewarded trials. Regressions include both Actor and Observer trials.

To evaluate the degree to which participants played the game rationally, we compared their behavior to that of an optimal “oracle” model (Figure 2). Specifically, we analyzed the decision to switch on the next trial as a function of three factors: (1) history of trial outcomes (including the current trial), (2) position of the current trial in the block, and (3) number of captured tokens in the current trial. To ensure a fair comparison between actor and observer, we first focus on *congruent trials* where both players chose the same arena.

### Behavioral consequences of outcome history (Figure 2a–c).

Because only one arena yields rewards, the oracle treats rewarded trials as confirmation of a correct choice and consecutive no reward trials as accumulating evidence for a switch (star, Figure 2a,b). The choice behavior of both humans and monkeys showed a similar pattern: Switch probability, denoted P(switch), was low after rewarded trials and increased monotonically with consecutive unrewarded trials (Figure 2a–b, S4a–b). This pattern was evident in single sessions (Figure 2c, left), remained significant in a logistic regression analysis that accounted for outcome history, trial in block, and number of captured tokens (Figure 2c, right), and was corroborated by the pattern of human confidence reports (Figure S3a).

### Behavioral consequences of trial position in the block (Figure 2d–f).

Since switches are less likely early on compared to later in the block, the oracle is more likely to switch later in the block (star, Figure 2d,e). As neither the oracle nor the participants were aware of block switches, we inferred subjective block switches from the behavior (Figure S2e–f) and registered trial position within subjective blocks (see Methods). In accordance with the oracle, P(switch) for both humans and monkeys increased monotonically with trial position in the block (Figure 2d–e, S4c–d). This effect was evident in single sessions (Figure 2f, left), remained significant in a multivariate logistic regression analysis (Figure 2f, right), and was corroborated by the pattern of human confidence reports (Figure S3a).

### Behavioral consequences of number of captured tokens (Figure 2g–i).

The probability of success increases with the number of captured tokens. Participants understood this contingency and aimed to maximize the number of captured tokens (see Methods; Figure S2g–h). Moreover, as predicted from the oracle (star, Figure 2g–h), participants must treat unrewarded trials with larger numbers of captured tokens as stronger evidence for a block switch. Qualitatively, this effect was evident in human participants’ behavior (Figure 2g, S3a), and to a lesser degree in monkeys (filled, Figure 2h, S4e–f). However, a more rigorous multiple regression analysis indicated that the effect was significant only in humans, and in Actor condition for one of the animals (Figure 2i, S3b).

### Behavioral consequences of the choice incongruence (Figure S5).

We also analyzed incongruent trials where participants chose opposite arenas. These trials were more frequent later in the block (Figure S5a,d), as expected by the higher switch probability, and were associated with lower confidence reports in humans (Figure S5c). Moreover, across human participants and one monkey, the actor was more likely to switch following incongruent trials (Figure S5b,e–g), indicating that players were influenced by each other’s decisions on top of their experiences and outcomes.

## Humans and monkeys discount observational evidence

By design, the oracle evaluates actor and observer trial outcomes identically (star, Figure 2j–k). In contrast, humans and monkeys weighted observational evidence less than experienced evidence (Figure 2j–k) even though actor and observer were randomly assigned and had identical visual experiences. This asymmetry was stronger in monkeys, possibly because the actor monkey received juice reward. Notably, the asymmetry was evident in single sessions (Figure 2l, left), and remained significant after accounting for other experimental factors (Figure 2l, right). This result indicates that humans and monkeys were less responsive to unrewarded trials as an observer. Notably, this asymmetry was not due to players spending less time looking at the screen when designated as observer (Figure S1e–m). This asymmetry was also reflected in humans’ confidence reports when they chose to stay in the same arena (Figure S3a).

## Experiential and observational evidence integration in the cingulate cortex

Our behavioral results provided compelling evidence that monkeys, like humans, integrate experiential and observational evidence to infer latent state switches. To investigate the underlying neural computations, we recorded neural activity in ACC (see Table S1 for stereotaxic coordinates) simultaneously from the two animals.

**Figure 3.**
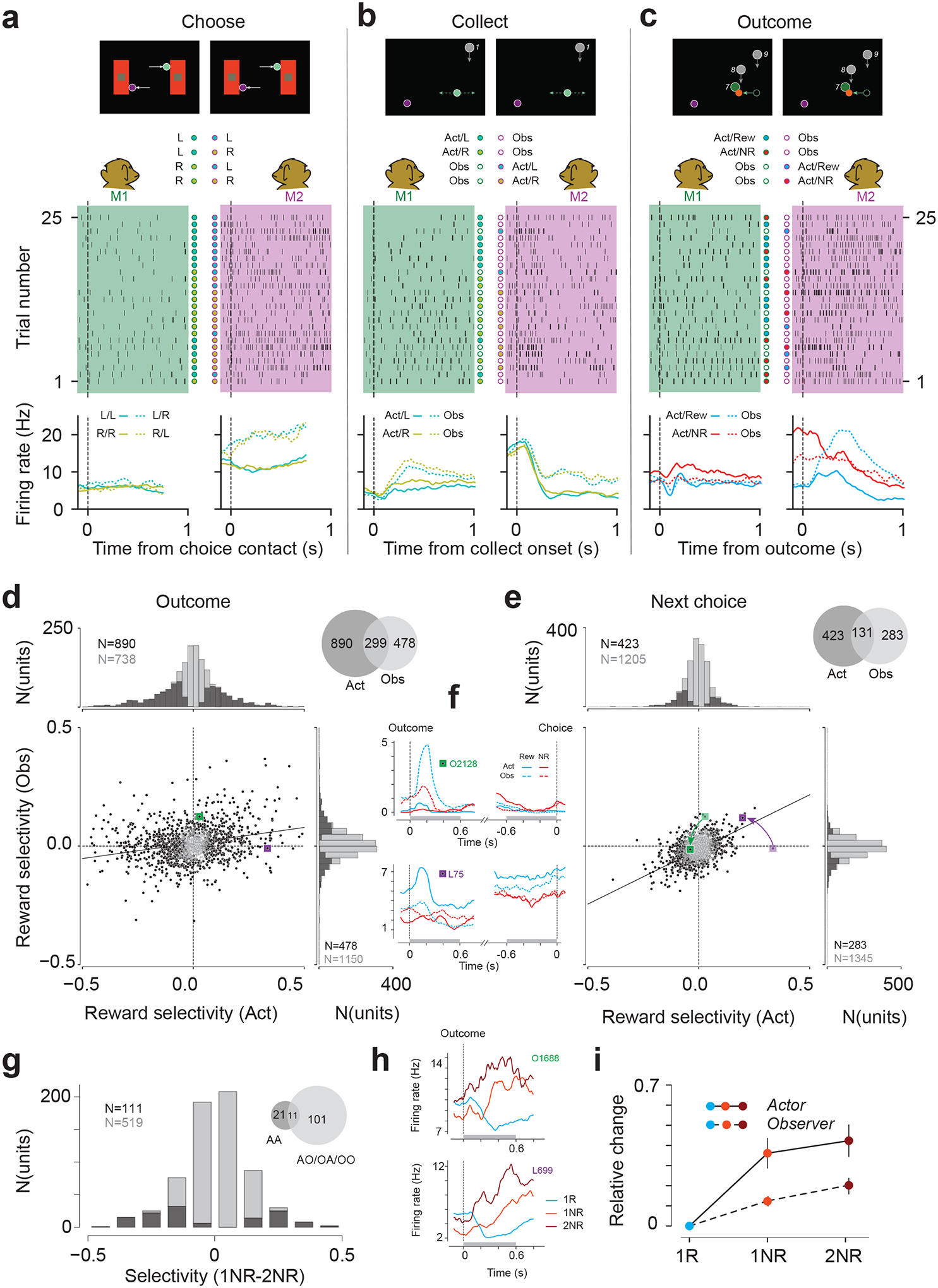
ACC neurons encode and integrate actor and observer outcome (**a**) Top: screen display at choice contact. L/R: choice of each monkey. Filled (open) circle indicates actor (observer). Middle: raster plot of action potentials recorded from a unit in M1 (left, green shade) and M2 (right, magenta shade). Dashed vertical line: choice contact. Trials are chronologically ordered from 1 to 25. Bottom: firing rate histogram of each unit. (**b-c**) Same as (**a**) with the same two units for the collect (**b**) and outcome (**c**) phase. (**d**) Reward selectivity after outcome, data pooled across sessions. Scatter: actor (x) vs. observer (y) selectivity (see Methods). Black line: regression. Black dots indicate significant selectivity in either condition. Top left and right: histograms show significant reward selectivity (actor: 890/1628; observer: 478/1628, permutation test, 1000 times, P<0.05). Top right: number of reward selective neurons. (**e**) Same as (**d**) before choice contact. (**f**) Firing rate histograms of example neurons as shown in (**d**) and (**e**). Top: Neuron from M1. Gray bars correspond to the 600 ms windows following outcome or before choice used for analysis in (**d**) and (**e**). Bottom: example neuron from M2. (**g**) Histogram of accumulation selectivity (see Methods). Black bars: neurons with significant selectivity in any of the four conditions (111/630; permutation test, 1000 times, P<0.05). (**h**) Firing rate histogram of example neurons with significant accumulation selectivity. Top: example neuron from M1. Dashed line aligned to outcome. Bottom: example neuron from M2. (**i**) Relative change in z-scored firing rate in the 600 ms window following outcome feedback between 1R and 1NR or 2NR. Solid line corresponds to actor conditions in 1NR and 2NR, dashed line corresponds to observer conditions. Data are presented as mean values +/− 95% confidence interval from bootstrap, N=1000.

Our recordings (M1: 31 sessions; M2: 20 sessions; simultaneous recording in 19 sessions) yielded 1628 units (M1: 859; M2: 769). Most task-modulated neurons were sensitive to multiple variables and their sensitivity could change throughout the trial (Figure 3a–c). For example, we found mixed selectivity to trial outcome for the actor and observer (Figure 3d,e,S6a–d) with a large proportion sensitive to actor outcome (bootstrap test, p<0.05 in 890/1628=54.7% of all neurons in both animals, see Methods), a smaller proportion to observer outcome (bootstrap test, p<0.05 in 478/1628=29.4% of all neurons in both animals), and a sizeable overlap between the two (299/1069=28.0% of outcome selective neurons). Moreover, this sensitivity changed throughout the trial, as evident from single neurons (Figure 3f) and across the population (Figure 3d–e, regression slopes). Notably, the alignment for outcome encoding between the actor and observer increased in the choice phase compared to the outcome phase (Figure 3d–e; regression slope in choice: 0.46±0.03; outcome: 0.13±0.01; variance explained in choice: 32%; outcome: 6%). This result is consistent with a gradual integration of distinct actor- and observer-dependent responses into an identity-agnostic outcome representation.

We further analyzed single neurons for evidence of outcome integration across actor and observer trials. Integration requires firing rate modulations for the actor and observer to be in the same direction; opposite directions would counter integration. Accordingly, we restricted our analysis to 630 neurons with same-sign outcome selectivity for actor and observer (Figure 3d–e, 1^st^ and ^3rd^ quadrants) and quantified the difference between firing rates in the 1NR and 2NR conditions (Figure 3g, S6e,f). Across this population, 17.6% (111/630) were more strongly modulated for 2NR compared to 1NR trials. This effect was also evident in the average firing rates of individual neurons (Figure 3h).

Previous work has shown that ACC neurons integrate cross-trial evidence in single-player tasks that involve only experiential evidence^3,10,23^. As such, it is critical to subdivide 2NR trials and distinguish between Actor-Actor trials that involve only experiential evidence and the other three conditions that have at least one observer trial (Actor-Observer, Observer-Actor, and Observer-Observer). Doing so, we found that 91.0% (101/111) of neurons that featured evidence accumulation were sensitive to observer trials (Figure 3g, inset).

A notable feature of behavior was the asymmetry in evidence accumulation: unrewarded trials, matched in every other aspect, were weighed more strongly in actor trials compared to observer trials. We therefore asked whether firing rate changes in neurons featuring evidence accumulation were also stronger for actor trials. Indeed, firing rate modulations in the 1NR and 2NR trials compared to rewarded trials were stronger in the Actor condition (Figure 3i, S6h–i, mean z-scored rate change in actor condition: 0.36 in 1NR, 0.42 in 2NR; observer condition: 0.12 in 1NR, 0.20 in 2NR; p<0.001 between Actor and Observer conditions, paired t-tests).

## The neural geometry of multi-agent evidence accumulation

While single neurons encoded a wide range of task variables, this sensitivity was typically mixed, changing both during the trial (Choose versus Collect versus Outcome phases) and as a function of trial type (e.g., Actor versus Observer). This property, which is common across frontal cortical neurons^46^, motivated further neural analysis at the population level, which can offer complementary computational insights^47–49^.

### A stable dimension encoding switch belief (Figure 4a–d).

**Figure 4.**
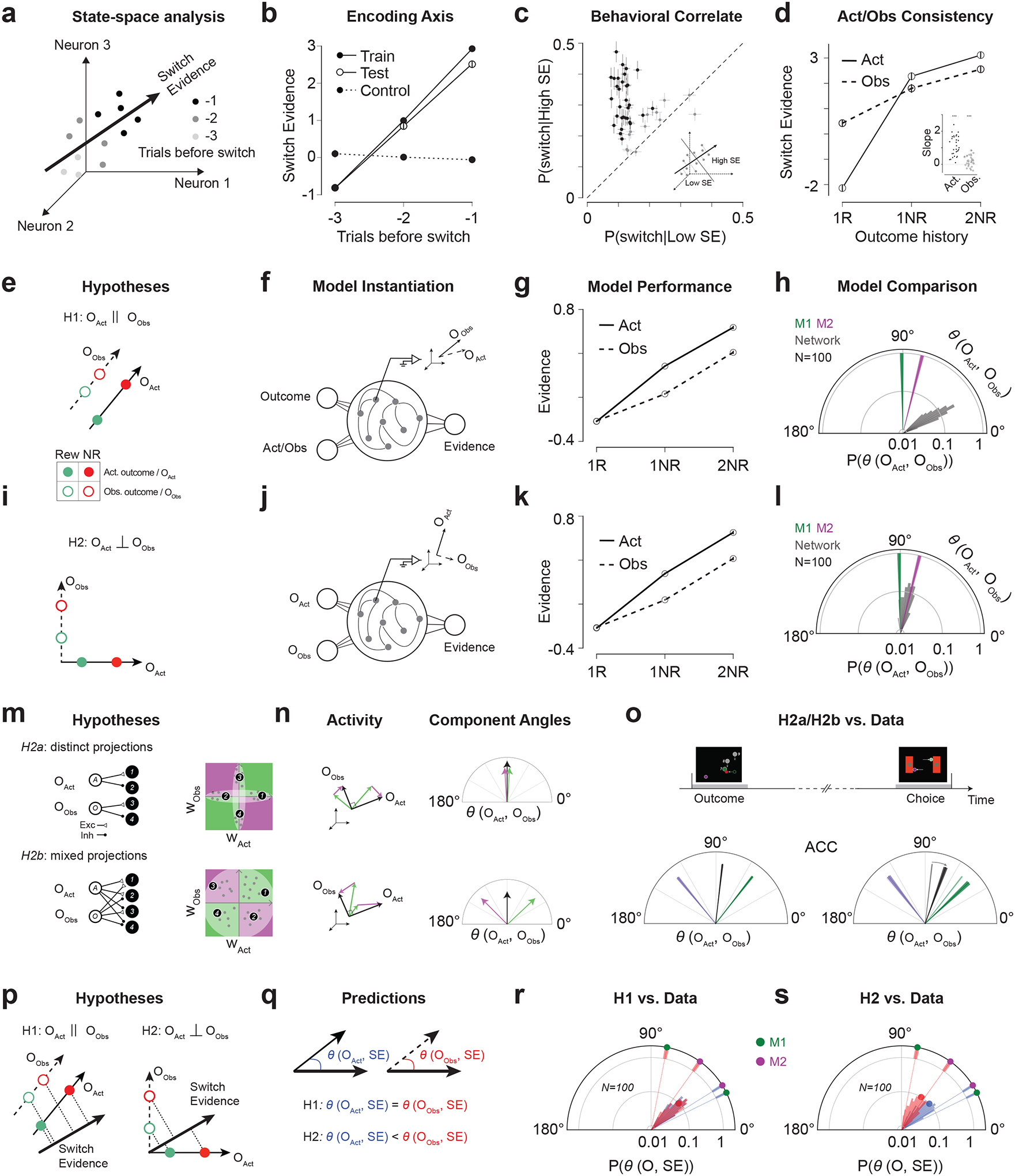
Population geometry of multi-agent evidence integration (**a**) Switch evidence dimension (SE) from regression on trials before switch. (**b**) Projection on SE relative to trials before switch for training (solid), test (open), and null control (dashed). (**c**) Proportion of switch trials conditioned on a low (x axis) or high (y axis) projection on SE for each session. Error bars: SEM. Black dots: significant differences (N=31/43 sessions, rank sum test, P<0.05). Inset: trials are sorted by SE projection relative to the session median. (**d**) Projection on SE by behavioral condition. Solid (dashed) line: Actor (Observer) conditions. Inset: slopes of regression for projection on SE over outcome history. (**e**) Parallel hypothesis on the organization of neural activity. (**f**) RNN instantiating the parallel hypothesis. (**g**) Performance of trained RNNs in (**f**). Solid (dashed) line represents output in actor (observer) condition. Error bar: 95% confidence interval. (**h**) Angle between actor/observer outcome dimensions (θ(O_Act_, O_Obs_)). Gray: from network activation over 100 model instantiations. Green (magenta): from ACC neural activity in M1 (M2) over 100 randomly selected halves of trials, width indicates standard deviation. (**i**-**l**) Same as (**e-h**) for the orthogonal hypothesis. (**m**) Network architecture hypotheses. Left: schematic of weights between input populations representing actor/observer outcome (A and O) and ACC neural population. Right: input weights for one neuron. (**n**) Outcome dimensions (black) decomposed into aligned (green) and anti-aligned (magenta) subspaces. (**o**) Decomposition of θ(O_Act_, O_Obs_) in ACC. (**p-q**) Geometry of outcome to switch evidence integration and outcome representations in H1 and H2. (**r**) Center blue/red angular distribution shows values of θ(O_Act_, SE)/ θ(O_Obs_, SE) for H1-instantiating RNNs. Blue/red circles: mean values. Dashed lines: ACC data, separately for the two animals (M1: green; M2: magenta). Shade indicate std values computed from randomly selected halves of trials, repeated 100 times. (**s**) Same as (**r**) for H2, with network data from (**j**).

First, we identified a dimension along which activity increased monotonically across trials leading to a behavioral switch (Figure 4a, see Methods). Using cross-validation, we verified that this dimension predicted switch behavior (Figure 4b; Figure S7a–b for individual animals). Importantly, this effect was specific to trials preceding switches and was not due to a trial-order effect (Figure 4b, dashed). We also confirmed the link between the encoding dimension and switch behavior by verifying that large and small projections of neural activity on the encoding dimension corresponded to high and low values of P(switch), respectively (Figure 4c).

Because behavior was influenced by both Actor and Observer trials, we examined projections onto the encoding dimension for each trial type separately. This dimension carried outcome information for both, with larger projections in 2NR than 1NR trials (p < 0.001 in both Actor and Observer conditions, paired t-tests; Figure 4d; Figure S7e–f for individual animals). This observation was evident within sessions as quantified by the regression slopes relating projections to the number of unrewarded trials (Figure 4d, inset). Consistent with behavioral asymmetry, regression slopes were steeper for Actor than Observer trials (p < 0.001, paired t-test, Figure 4d, inset). Control analyses confirmed that this difference was not explained by unequal trial counts or neuron numbers (Figure S11a–c).

### Identity and outcome inputs to ACC (Figure 4e–l).

We examined the neural state space to characterize how inputs to ACC convey information about identity and outcome. We considered two hypotheses. **H1** proposes a shared, identity-agnostic input for outcomes and a separate input for identity. This arrangement yields parallel encoding dimensions for Actor and Observer outcomes (Figure 4e). **H2**, in contrast, posits independent inputs for experienced and observed outcomes, producing orthogonal encoding dimensions (Figure 4i).

To test these hypotheses, we compared the geometry of ACC population activity with that of recurrent neural network (RNN) models implementing each hypothesis. In **H1**, the RNN received one input conveying identity-agnostic outcome information and another signaling identity (Figure 4f). In **H2**, it received separate inputs for experienced and observed outcomes (Figure 4j). We trained 100 randomly initialized RNNs per hypothesis (see Methods).

All RNNs successfully integrated evidence and reproduced the behavioral asymmetry (Figure 4g,k; Figure S8). We then compared their outcome representational geometry to that of ACC, identifying Actor and Observer encoding dimensions in each dataset and evaluating H1 and H2 based on the angle between these dimensions in the models and ACC.

We compared RNNs instantiating H1 and H2. The angle between Actor and Observer dimensions was smaller and closer to parallel in H1 (30.77 ± 6.88°, mean ± SD, N = 100; Figure 4h) and larger, approaching orthogonality, in H2 (78.89 ± 7.56°, N = 100; Figure 4l). ACC data showed similarly large angles (M1: 90.83 ± 1.35°; M2: 75.97 ± 1.28°; N = 100 splits per animal), consistent with H2 predictions (Figure 4h,l). This orthogonality was not explained by firing rate differences in rewarded trials: differences during unrewarded trials were equally strong and frequent (rewarded: n=375/1628, p<0.05, average z-scored rate difference=0.31; unrewarded: n=358/1628, average z-scored rate difference=0.34; Figure S12c). Moreover, orthogonality persisted when considering only unrewarded trials (Figure S12f).

To test whether the independent inputs are necessary for orthogonality, we trained additional RNNs with the H1 input architecture but imposed a constraint enforcing a large angle between the Actor and Observer outcome dimensions. These models failed to perform the task (Figure S13). These findings strengthen the hypothesis that ACC receives Actor and Observer outcome information via independent input pathways.

### Input projection patterns onto ACC (Figure 4m–o, S9a–c).

We analyzed population responses in ACC to dissect the organization of input projections onto ACC. Our previous analysis indicated that ACC receives independent inputs associated with actor and observer outcomes. This orthogonality is consistent with two hypotheses, denoted H2a and H2b. H2a posits that the actor and observer inputs project to disjoint ACC subpopulations (Figure 4m, S9a). This organization is consistent with the input orthogonality because disjoint populations are inherently orthogonal. H2b, in contrast, posits mixed projections to the same population in ACC (Figure 4m, S9a), which could also result in orthogonality.

Many neurons were sensitive to both actor and observer outcomes (Figure 3d, S6a–b). This result provides evidence for some level of mixed projection. To distinguish between H2a and H2b more definitively, we divided the neurons into two groups. The first group, which we refer to as aligned, include neurons whose firing rates move in the same direction for actor and observer conditions, either positively or negatively (Figure 4m, S9a green). The second group, which we refer to as anti-aligned are the neurons that encode outcome with opposite signs (Figure 4m, S9a magenta).

Critically, H2a and H2b make distinct predictions about the geometry of activity within the subspaces formed by these groups. In H2a, where projections are disjoint, the angles between actor and observer outcome representations remain orthogonal for both aligned and anti-aligned neurons (Figure 4n, S9b–c). In contrast, H2b predicts a divergence in the angle relationships: for aligned neurons, the encoding dimensions are more aligned, resulting in acute angles, whereas for anti-aligned neurons, the encoding dimensions are oppositely oriented, leading to obtuse angles (Figure 4n, S9b–c).

In ACC, at the time of outcome, the decomposed angle between actor and observer dimensions had large positive and negative components, consistent with H2b – not H2a (Figure 4o; Figure S7g–h for individual animals). As the task proceeds to time of choice for the next trial, the two vectors become more aligned (angle at outcome: 83.46° ± 0.92°, choice: 69.94° ± 1.98°, Figure 4o), consistent with the increased correlation of single neuron selectivities (Figure 3e). Together these results suggest that the actor and observer outcome inputs drive ACC through mixed projection patterns.

### Geometry of evidence integration (Figure 4p–s).

For effective integration, the switch evidence dimension should be outside the null space of both actor/observer outcome encoding dimensions. This predicts that the angle between the actor outcome and switch evidence, *θ(O*_*Act*_*,SE)*, as well as the angle between the observer outcome and switch evidence, *θ(O*_*Obs*_*,SE)* must be less than 90 deg. However, the relative geometry of these dimensions differ under the two hypotheses (Figure 4p). Under H1, because the outcome encoding dimensions are parallel, *θ(O*_*Act*_*,SE)* and *θ(O*_*Obs*_*,SE)* must be the same (Figure 4p). In contrast, under H2, because the outcome encoding dimensions are orthogonal, *θ(O*_*Act*_*,SE)* and *θ(O*_*Obs*_*,SE)* can assume different values (Figure 4q).

We measured *θ(O*_*Act*_*,SE)* and *θ(O*_*Obs*_*,SE)* in both the models and ACC (see Methods). The representational geometry in ACC was better captured by H2 (Figure 4r,s). In both ACC and H2-instantiating RNNs, *θ(O*_*Act*_*,SE)* was smaller than *θ(O*_*Obs*_*,SE)* (RNN_H2_: 43.15±10.22 vs. 59.11±10.89 deg., M1: 26.25±0.88 vs. 79.05±1.42 deg., M2: 31.96±0.94 vs. 54.40±1.35 deg.). This difference was not explained by unequal trial counts or numbers of outcome-responsive neurons and persisted after controlling for both factors (M1: 34.07 ± 1.82 vs. 74.05 ± 2.44 deg., M2: 33.20 ± 1.37 vs. 48.88 ± 1.75 deg. Figure S11d). By contrast, the two angles had similar magnitudes in the H1-instantiating RNNs (44.03 ± 7.35 vs. 43.48 ± 7.80 deg.).

For integration to occur, actor and observer outcomes must drive a common switch evidence dimension. In this respect, the smaller *θ(O*_*Act*_*,SE)* relative to *θ(O*_*Obs*_*,SE)* may provide a neural explanation of the behavioral asymmetry in experiential versus observational evidence integration. Specifically, if integration occurs by linear projection of activity along the outcome dimension onto the switch evidence dimension, then a smaller angle between the actor outcome and switch evidence dimensions would enable the same strength of evidence collected on actor trials to produce a higher increment in cumulative evidence (Figure 4p).

To further substantiate the relationship between neural geometry and behavioral sensitivity, we computed the outcome-switch angle separately for each session, for both Actor and Observer conditions. Pooling the data across the two animals, we found strong support for our hypothesis: there was a significant negative correlation between behavioral sensitivity and neural angle across sessions (Figure S14a–c).

Additional analyses of these variables for the two animals and the two conditions separately (Figure S14d–i) revealed that the effects were significant for the Actor condition in monkey M1 and the Observer condition in M2 only. Based on this finding, we hypothesized that the two animals were engaged in a leader–follower dynamic with M2 being more strongly influenced by M1 than the other way around, which was borne out of additional behavioral analyses (Figure S14j; also see Figure 2l).

Together, these results suggest that the angles between switch evidence and outcome dimensions in ACC account for the asymmetry in behavioral sensitivity between Actor and Observer conditions.

So far, we configured all RNNs such that their output weights were fixed. To test the effect of this assumption on our findings, we performed control analyses on RNNs built with learnable output weights (Figure S10b,d). The geometry of evidence integration in these readout-learnable networks was different from both the readout-fixed networks and the ACC. Specifically, they exhibited relatively higher alignment between actor and observer outcome dimensions and outcome and evidence dimensions (Figure S10b,d). These results suggest ACC internal dynamics and not its downstream projections are responsible for evidence integration.

## Discussion

Our work brings together two important yet traditionally distinct areas of research concerning the role of ACC in cognition. One important function of ACC is to monitor and integrate one’s experience over time to inform strategic decision-making^6,11,17^. This function supports a wide range of mental computations including explore-exploit trade-offs, cost-benefit analysis, conflict monitoring, and causal inference^1–4,19,23,25^. Another function ascribed to ACC is sensitivity to observed reward and punishment enabling vicarious learning^35,36,39–42^. Our work offers a unifying perspective wherein ACC plays a general role in integrating experiential and observational outcomes over flexible timescales to update belief about environmental states. The confluence of these two research directions brings to focus several important questions.

First, what anatomical substrates and circuit motifs enable the integration of information about self and other? We found that ACC encodes all three key computational variables needed for integration: actor outcome, observer outcome, and integrated belief. A comparison of population activity between models and ACC provided evidence that actor and observer outcomes were associated with activity patterns in orthogonal subspaces. This finding suggests that ACC computes beliefs about the state of the environment by integrating outcome information from distinct identity-dependent input streams.

Analysis of the geometry of neural representation is often used to infer computational algorithms. For example, subspace orthogonality is thought to prevent interference and maximize robustness^47,50–52^, and factorized representations are thought to facilitate structural generalization^49,53–57^. In our work, we augmented this analysis with single-neuron tuning properties to dissect the organization of input projections onto ACC. Results indicated that ACC did not rely on disjoint subpopulations for actor and observer information. Instead, actor/observer information was supplied via overlapping projections. We do not know the constraints that determine the organization of these projections. However, in our experiment, this mixing may facilitate the integration process. With disjoint subpopulations, the integration would have to be augmented by a gating mechanism to select the subpopulation that has to be integrated on each trial. In contrast, the mixed representation provides a single subpopulation of outcome-aligned neurons that can be used for integration in all trials.

Our analyses indicated that ACC coding properties changed throughout the inter-trial interval (Figure 4o, S15)^58^. One notable feature was the reduction of the angle between the population vectors encoding the actor and observer outcomes, from the outcome phase to the choice phase in the next trial. This finding is reminiscent of prior work showing high-dimensional firing-rate vectors rapidly decay to a single dimension during the process of decision-making^59^. However, in our work, this process unfolded in the presence of multiple inputs (two agents) and long timescales (across trials), which pose important constraints on the circuits responsible for evidence accumulation in ACC^60.61^.

Second, does the brain process experiential and observational information similarly? In our two-player game, although humans and monkeys integrated experiential and observational outcomes, they learned less from observations. Discounting observational evidence has been reported previously^43–45^. However, several aspects of our study reinforce the view that there is a fundamental asymmetry between learning from experience and observation. By interleaving actor and observer trials while collecting each player’s choice on every trial, we could track both players’ evolving beliefs with precision. This design choice as well as our analysis of congruent and unrewarded trials enabled us to rule out various confounds that could lead to this asymmetry. Finally, we found this asymmetry to be stronger in monkeys, possibly because monkeys received juice reward, which could accentuate the difference between experience and observation. The difference between humans and monkeys may also stem from superior social cognition in humans enabling more effective evaluation and integration of observations.

We identified a neural correlate of this asymmetry in the ACC, where signals encoding actor outcomes were more closely aligned with cumulative switch evidence than those encoding observer outcomes. Validating the functional relevance of this finding will require precise patterned activations of subpopulations of neurons in ACC^62–64^. Additionally, characterizing the behavioral contingencies and neural constraints that give rise to this asymmetry remains an important direction for future research.

In sum, our work establishes the basic mechanisms of multi-agent evidence integration and offers a starting point for addressing exciting and unresolved questions about social learning. Extensions of our work can be used to study the mechanisms through which cognitive factors such as belief about the partner’s skill level, their prior knowledge about task contingencies, and their social rank influence observational learning.

## Methods

We collected behavioral data from humans, and behavioral and neurophysiological data from rhesus macaque monkeys (Macaca mulatta). Experimental procedures for humans were approved by the Committee on the Use of Humans as Experimental Subjects at the Massachusetts Institute of Technology. Experimental procedures for animals conformed to the National Institutes of Health guidelines and were approved by the Committee of Animal Care at the Massachusetts Institute of Technology.

### Experimental procedures for non-human primates

Two monkeys (M1, female, 6 Kg, aged 6; M2: male, 11 Kg, aged 11) were seated comfortably in two adjacent primate chairs in a dark quiet enclosure at a distance of 40 inches. Animals were head-restained, facing forward, and unable to see one another. Stimuli were presented on two side-by-side display monitors (Acer R240HY) 40 inches apart (center to center), at a normal distance of 19 inches from the animals’ eyes. The monitors displayed identical stimuli throughout experiments. Each animal could manipulate a joystick (Logitech Extreme 3D Pro) placed at a distance adjusted for each animal’s reach (~6 inches) in front of their chair. Joysticks were physically constrained to left/right movements only. The joystick digital output (1–1024) was thresholded to three states: left movement (1–463), no movement (464–560), and right movement (561–1024). Eye movements were sampled at 1kHz using infrared cameras (Eyelink 1000, SR Research). The MWorks software package (http://mworks-project.org) and MOOG library^65^ (https://jazlab.github.io/moog.github.io/) were used to generate visual stimuli and to enforce behavioral contingencies. A photodiode was used to sync electronic events with stimulus presentations.

Neural recordings were made from the anterior cingulate cortex (ACC) with 64-channel linear probes with 50-μm inter-electrode spacing (V-probe, Plexon Inc.) inserted through a rectangular recording chamber. Extracellular signals were bandpass filtered (300 Hz to 6 kHz) and digitized (sampling rate: 30 kHz) using two 32-channel headstages (Intan Technologies), and collected using OpenEphys software (http://www.open-ephys.org). Spike sorting and curation were carried out using Kilosort 3 (https://github.com/MouseLand/Kilosort) and phy (https://github.com/cortex-lab/phy). Recording sites and number of sessions/trials are reported in Table S1. Data analysis was performed using custom Python code.

### Experimental procedures for humans

We recruited a total of 14 participants. All participants gave informed consent, were naive to the purpose of the study, and had normal or corrected-to-normal vision. Participants were asked to play the single-player version of the task first, and after gaining familiarity were invited to play the two-player version. One participant did not learn the task after three single-player sessions (set to be an exclusion criterion before data collection begins). Three participants withdrew voluntarily after finishing the single-player sessions. The remaining 10 participants (6 males and 4 females, aged 18–65) completed the two-player sessions. These participants were divided into 5 fixed pairs (1 female-female, 2 male-male, and 2 male-female). Each session lasted ~60 min. Participants were paid a fixed amount at the end of each session. Those who finished all single and two-player experiments were paid an additional 50% of all their earnings as a bonus.

In each session, participants sat in adjacent dark enclosures, separated by an opaque curtain to prevent visual contact. Each enclosure had identical equipment (monitor, keyboard, joystick connected to a Mac mini). Participants knew their monitors displayed the same visuals, though they couldn’t see the other screen. In the single-player experiment, only one setup was used. Like monkey experiments, tasks, stimuli, and behavioral contingencies were controlled by MWorks and MOOG software packages.

### Behavioral task

We devised a two-player trial-based game. Each trial consists of two hierarchically organized phases. Phase 1 begins with the presentation of the two arenas on the two sides of the monitor and two disks stacked on top of one another mid-way between the two arenas representing the two players (‘avatars’). Each arena is a red rectangle (8.2 × 16.4cm) with a central gray square (width: 4.1cm) placed 3 cm to the left or right of the vertical midline. Each avatar appears as a yellow disk (diameter: 2.4cm) placed 3.85 cm above or below the horizontal midline. After 750 ms, the two yellow avatars are randomly assigned to the two players by a change of color, purple for player 1, and green for player 2. At this time, players must choose either the left or right arena by moving their avatar in the direction of their preferred arena. Once an avatar contacts an arena, the joystick control for that avatar is temporarily relinquished. After both players choose their preferred arena an additional 750 ms interphase-interval delay is imposed and then the task moves to the second phase. In phase two, players are randomly assigned to be the actor and observer, the red rectangles disappear, and the two avatars are displaced from the edge to the bottom interior of the corresponding arenas. The actor is placed 10.1 cm away from the vertical midline and 6 cm below the horizontal midline. The observer is placed 10.1 cm away from the vertical midline and 12 cm below the horizontal midline. Immediately afterward, the actor starts playing the token capture game on its monitor, a copy of which is shown on the observer’s monitor. The observer was free to move the joystick, but joystick movements were disconnected from avatar control during Observer trials. The actor must use the joystick to move the avatar left or right to capture 15 falling tokens (gray circular disks of diameter: 6.72 cm). The tokens drop sequentially every 1/3 s, starting 16.8 cm above the center and moving directly downward at the constant speed of 50.4 cm/s. The horizontal positions of tokens are sampled from a Gaussian Process (GP) centered at the initial position of the actor’s avatar with a squared exponential kernel. To vary task difficulty across trials, the standard deviation of the GP was sampled from a discrete uniform distribution ([2.52, 5.04, 7.56] cm).

Each trial ends in either a win or a lose state. To maximize wins, the actor must select the correct arena and collect as many tokens as possible. The correct arena (the one associated with a non-zero win probability) switches covertly in a blocked fashion (see below). In either arena, captured tokens turn green, while missed tokens disappear without effect. A win is signaled by a change of the color of the avatars and an auditory tone. A trial ends either with a win or after all 15 tokens pass. The observer receives no reward but can monitor trial progression and infer the outcome from the visual and auditory feedback. Each trial lasts 5.42 ± 2.18 seconds. After the trial, the display remains stationary for 1 second before switching to a uniform black screen for an inter-trial interval of 2.676 ± 0.085 seconds.

Before the two-player game, players completed a single-player version with a single avatar, designated as the actor. All other task aspects remained identical to the two-player version. The task contingencies were identical for humans and monkeys with a few exceptions as follows.

#### Non-human primates.

(a) The switching probability is 0 for the first 10 trials, 1/3 for trials 11–24, and 1 for trial 25. (b) In the correct arena, captured tokens may trigger a win with a 15% probability. (c) A win triggered a juice drop for the actor. (d) Animals first played the single-player version until their performance stabilized. The single-player data reported are from sessions with stable performance. (e) They were then introduced to the two-player version without electrophysiology recordings until performance stabilized. Reported behavioral and neural data are from subsequent sessions with simultaneous behavioral and physiological recordings.

#### Humans.

(a) The switching probability is 0 for the first 10 trials, 1/3 for subsequent trials. (b) In the correct arena, captured tokens may trigger a win with a 10% probability. (c) Before the start of the first session, participants read verbal instructions about the game but were not informed of the reward probabilities or state-switching statistics. (d) Participants played the single-player version for 5 or 6 sessions (see below). Single-player sessions started with 50 practice trials where the correct and incorrect arenas were cued (green and yellow, respectively). After an optional short break, participants completed 600 trials of the one-player game with 2-minute breaks every 200 trials. Reported single-player data are from sessions 1–5 and do not include the practice trials. (e) Initially, participants were scheduled for five single-player sessions. During data collection, a confidence-reporting step was added, requiring participants to use a keyboard to report their confidence on every trial (1: “not confident at all” to 4: “fully confident”) after selecting the arena, in both single and two-player games. Nine participants who completed five sessions of single player in the original task (i.e., with no confidence report) played a sixth session with confidence reporting, while the tenth participant used the augmented task from the start and did not require a sixth session. (f) Participants received instructions about the two-player game before the first two-player session. Each session included 525 trials with two 2-minute breaks after every 175 trials. Each pair completed 15 two-player sessions, totaling 7875 trials.

### Analysis of behavior

We used players’ choice behavior to quantify the probability of choosing the correct arena from 4 trials before to 10 trials after a block switch (Figure 1h–k).

#### Solipsistic agent.

To test if a player was sensitive to observer trials, we compared its performance to the performance predicted if the player were to ignore all observer trials and treat the two-player game as a single-player. We refer to this hypothetical player as *solipsistic*. If we denote a win state at trial *n* by *R*_*n*_, then the average probability of *R*_*n*_ for a solipsistic actor in the two-player game (*P*_*2p*_) based on their average performance in the one-player game (*P*_*1p*_) can be written as follows:

Eq. 1 P2pRn=∑0nnk(12)nP1p(Rn-k)

The <.> denotes average probabilities. The sum runs over the Observer trials preceding trial *n* since the last block switch, indexed by *k*. To understand this equation, consider a sequence of *n* trials with *k* Observer and *n-k* Actor trials. The binomial coefficient (*n*-choose-*k*) counts such combinations, while *(½)*^*n*^ gives their probability, with *(½)*^*k*^ for *k* Observer and *(½)*^*n-k*^ for *n-k* Actor trials. The term *P*_*1p*_*(R*_*n-k*_*)* implements the assumption that the actor disregards Observer trials, behaving as if only *n-k* trials have passed since the last block switch.

We tested the significance of the difference in accuracy between this agent and the participant using t-test on the average P(correct) for positions [0,10].

#### Oracle agent.

To estimate an upper performance bound on trial *n*, we simulated an oracle agent who mimicked the player’s choices for trial *1:n-1* but selected the correct choice on trial *n*. Therefore, if the correct choice on trial *n* is to switch, regardless of whether trial *n–1* was rewarded or not, the oracle will switch. This results in a non-zero probability of switching after rewarded trials.

### Difficulty of the collecting tokens

In the second phase, token positions were random, creating varying difficulty levels. To assess whether players attempted to maximize captured tokens, we analyzed performance on unrewarded trials as a function of difficulty (*D*), defined as the sum of absolute distances between successive tokens.

Eq.2 $$D=\sum_{0}^{14}\left|x_{i+1}-x_{i}\right|$$

Here, $x_{i}$ is the horizontal position of the i-th token in screen coordinates. Since each unrewarded trial has 15 tokens, the difficulty is the sum of 14 horizontal displacements.

### Subjective belief about the correct arena and block switches

Since the correct arena and block switches were covert, we developed a method to estimate participants’ subjective beliefs about the arena and trial position within a block. The first rewarded trial of a session was assumed to indicate the correct arena and was assigned position 1 of the first block. The position incremented until the first reward on the opposite arena, which was assumed to signal a block switch, resetting the position to 1. Early, Mid, and Late trials were defined as [1,5], [6,10], and [11, ∞), with bin sizes balanced as closely as possible.

### Regression analysis of switch behavior and confidence

We used logistic regression to quantify the dependence of switch behavior on various factors after unrewarded trials, including the number of consecutive unrewarded trials, the number of tokens captured, and position in the trial.

Eq. 3 $$\text{Switch}=\beta_{u}N_{\text{unrewarded}}+\beta_{t}N_{\text{tokens}}+\beta_{p}N_{\text{position}}+\beta_{0}$$

Switch is a binary variable indicating when the player chooses a different side than the actor’s current choice (i.e., the arena for which direct evidence is acquired). $N_{\text{unrewarded}}$ is the number of consecutive unrewarded trials in the same arena. We only included up to 4 consecutive unrewarded trials in this analysis. $N_{\text{tokens}}$ is the number of touched tokens, and $N_{\text{position}}$ is the subjective trial position in the block.

Combining both Actor and Observer trials, we used simple logistic regression (for each monkey) or mixed effects logistic regression (for human participants):

Eq. 4 $$\text{Switch}=\beta_{c}I_{\text{condition}}+\beta_{u}N_{\text{unrewarded}}+\beta_{t}N_{\text{tokens}}+\beta_{p}N_{\text{position}}+\beta_{0}$$

$I_{\text{condition}}$ is a binary indicator variable (0 for Actor, 1 for Observer).

We also computed the contribution from choice conflict for the Actor condition. For this analysis, we included incongruent trials, which were excluded in previous regressions.

Eq. 5 $$\text{Switch}=\beta_{u}N_{\text{unrewarded}}+\beta_{t}N_{\text{tokens}}+\beta_{p}N_{\text{position}}+\beta_{cg}I_{\text{congruence}}+\beta_{0}$$

$I_{\text{congruence}}$ is a binary indicator variable (1 for incongruent, 0 for congruent).

For human participants, we additionally performed mixed effects linear regression on their confidence report.


Eq. 6 $$\text{Confidence}=\beta_{c}I_{\text{condition}}+\beta_{u}N_{\text{unrewarded}}+\beta_{t}N_{\text{tokens}}+\beta_{p}N_{\text{position}}+\beta_{0}$$

### Analysis of single neurons

We estimated each neuron’s firing rate by averaging spike counts in shifting 100 ms time bins with a 10 ms step size. We analyzed firing rates aligned to different task events including the choice time (i.e., when the avatar contacts an arena) and outcome (i.e., reward time in rewarded trials and end of token collection in unrewarded trials). For visualization, binned firing rates were smoothed using a 3-bin moving average.

### Single-neuron sensitivity to outcome and choice

We measured selectivity to reward outcome in self/other conditions using receiver operating characteristic (ROC) analysis based on spike counts within 600-ms windows, either after the outcome or before the choice. The ROC score, calculated as the area under the performance curve of a binary classifier, classified trials as rewarded or unrewarded—either for the current trial (outcome) or the previous trial (choice). To center selectivity at 0, we subtracted 0.5 from the score, where values of −0.5 and 0.5 indicate perfect separation of reward outcome with lower or higher firing rates for the reward condition, respectively. Significance was assessed via bootstrap (1000 iterations, p<0.05). To compute the correlation of ROC scores between self and other conditions, we performed total least squares regression between self and other selectivities.

### Single-neuron sensitivity to cumulative errors

We computed selectivity for the history of congruent unrewarded trials (1NR vs. 2NR) using ROC analysis for neurons that exhibited significant reward selectivity in either the Actor or Observer condition and maintained the same selectivity sign in both. Binary classifiers were constructed for the four possible consecutive outcomes: 1NR-Actor vs. 2NR-Actor (AA), 1NR-Actor vs. 2NR-Observer (AO), 1NR-Observer vs. 2NR-Actor (OA), and 1NR-Observer vs. 2NR-Observer (OO). For neurons with significant selectivity in any condition, we also calculated the average differences in z-scored firing rates across rewarded Actor trials, 1NR (Actor or Observer), and 2NR (Actor or Observer). Neurons with consistent rate changes (either 1R<1NR<2NR or 1R>1NR>2NR) were classified as encoding cumulative error.

### Single-neuron sensitivity to Actor and Observer conditions

We compared trial-by-trial firing rates in the 600ms after outcome between Actor/Observer conditions using rank sum test, separately for rewarded and unrewarded trials. For neurons with significant differences, we calculated the absolute z-scored firing rate differences between Actor and Observer conditions.

### Population activity for actor/observer outcome and switch evidence

We used targeted dimensionality reduction (TDR) to identify encoding dimensions of actor outcome, observer outcome, and switch evidence^47^. To do so, we used regression to relate each neuron’s spike count within a 600 ms window following the outcome to different task variables.

For Actor/Observer outcome (analyzed separately), we used the following regression:

Eq. 7 $$Z=\beta_{\text{outcome}}I_{R/N}+\beta_{\text{choice}}I_{L/R}+\beta_{0}$$

$I_{R/N}$ is an indicator variable for outcome (1 for unrewarded, 0 for rewarded) and $I_{L/R}$ an indicator variable for choice (−1 for left, 1 for right). To reduce estimation noise, only neurons that had more than 5 trials in all conditions were included.

For Actor/Observer outcome without rewarded trials, we used the following regression:

$$Z=\beta_{\text{history}}I_{1NR/2NR}+\beta_{\text{choice}}I_{L/R}+\beta_{0}$$

$I_{1NR/2NR}$ is an indicator variable for history (0 for 1NR trials, 1 for 2NR trials).

For switch evidence, we used the following regression:

Eq. 8 $$Z=\beta_{\text{switch}}N_{\text{preswitch}}+\beta_{\text{choice}}I_{L/R}+\beta_{0}$$

$N_{\text{preswitch}}$ represents the distance in trials from the next switch, with values of −1 for one trial before, −2 for two trials before, and −3 for more than two trials before the switch. $I_{L/R}$ is an indicator variable for choice (−1 for left, 1 for right). To reduce estimation noise, only neurons that had more than 10 trials in all conditions were included.

After solving the regression for all neurons, we arranged the coefficients in a matrix and orthogonalized columns using QR-decomposition, requiring R to have positive diagonal values such that the columns of Q provided are orthogonalized set of coefficients for each variable.

For Actor/Observer (Equation 7), we used the orthogonalized coefficients associated with the outcome (first column) as the Actor/Observer outcome dimension for each condition. For the switch evidence (Equation 8), we used the coefficients associated with $N_{\text{preswitch}}$ (first column) as the switch evidence dimension. For cross-validation of the switch evidence dimension, we randomly selected one trial per condition, computed the evidence dimension from the remaining trials, and projected the held-out trial activity onto this dimension. This process was repeated 100 times, generating 100 cross-validated projections per condition.

### Predicting switch behavior from switch evidence dimension

We projected neural activity onto the switch evidence dimension to predict switch behavior in the next trial. For each recording session, we selected trials with at least two recorded neurons and at least 10 switch trials. Using spike counts from all but one randomly selected trial, we computed the switch evidence dimension and projected activity from all trials onto it, generating a distribution of projection values. The held-out trial was classified as high or low evidence based on whether its projection value was above or below the median of this distribution. This process was repeated 1000 times per session. We then calculated the proportion of switch trials in the high and low evidence groups, considering sessions significant if the low evidence group had a significantly lower proportion of switches, as determined by a rank-sum test.

### Contribution of actor/observer outcome to switch evidence dimension

We computed neural switch evidence in Actor and Observer conditions by randomly selecting one trial from each Actor/Observer × 1R/1NR/2NR condition as a held-out test trial, deriving the evidence dimension from the remaining trials, and projecting the test trial activity onto this dimension. Only accumulation-selective neurons from sessions where neural switch evidence significantly predicted switching behavior were included. This process was repeated 100 times to generate a distribution of projection values for each condition.

### The geometry of actor/observer outcome, and switch evidence

We measured pairwise angles between actor outcome, observer outcome, and switch evidence dimensions using a randomly selected half of the trials and repeated this process 100 times generating a distribution of angles. We also measured the angle between actor and observer separately in two subspaces: the aligned subspace, computed from those neurons whose coefficients in Actor- and Observer-outcome coefficients had the same sign; and the anti-aligned subspace, which had opposite signs.

### Stability of switch evidence in the inter-trial interval

We computed the switch evidence (SE) dimension using a sliding window of 600 ms with a 300 ms step size, over the 3 seconds following outcome onset. For each time window, we calculated the angle between the SE dimension at that time and the SE dimension computed immediately after the outcome.

### Analysis of neural geometry and behavior across sessions

We measured pairwise neural angles between outcome and switch evidence dimensions using data from individual sessions. To test whether these angles were predictive of learning rate, we performed a regression analysis relating neural angle to behavioral sensitivity, defined as the slope of the regression between P(Switch) and the number of unrewarded trials. We carried out this analysis across several data subsets: (1) Combined across animals and conditions (Actor and Observer); (2) Combined across animals but separately for Actor and Observer conditions; (3) Separately for each animal, combining across conditions; (4) Separately for each animal and each condition.

Because sessions varied in the number of recorded neurons, we used weighted total least squares regression, assigning weights based on the expected variance in angle estimation. Specifically, we estimated each session’s weight using the following procedure:

We first identified the session with the maximum number of neurons (N) and computed its outcome-switch angle (θ). This session was used as the reference and assigned a weight of 1. To estimate the reliability of angle measurements in sessions with fewer neurons (M < N), we simulated how angle estimates degrade with reduced dimensionality. We constructed two vectors in N-dimensional space with a known angular separation θ, then repeatedly (5000 times) subsampled M random dimensions and computed the angle between the truncated vectors. This yielded a distribution of estimated angles for dimensionality M. The variance of this distribution reflects the expected noise in angle estimation for a session with M neurons. We then used the inverse of this variance as the weight assigned to that session in the regression.

### Session level analysis of neural geometry and behavior

We measured pairwise angles using data from single sessions. To assess the correlation between a neural angle (between outcome and switch evidence dimensions) and rate of learning (the regression slope of P(Switch) over number of unrewarded trials), we performed weighted total least squares regression between the neural angle and the behavioral slope for Actor and Observer conditions. The weight of each session was determined by the expected variance of measurement given the number of neurons available in that session, relative to the maximum number of neurons recorded in any session. Given the session with the maximum number of neurons N and measured angle *θ*, we constructed two vectors V1 and V2 in N-dimensional space with *θ* between them. For this session the weight was set to 1. For each session with number of neurons M<N, we subsampled V1 and V2 in randomly selected M dimensions from the original N-dimensional space, and computed the angle between them. We repeated this process 5000 times to obtain the standard deviation SD(M). The inverse of this variance was used as the weight in the regression.

To assess the degree to which either animal’s learning rate as an observer was correlated with the actor’s, we performed total least squares regression between the behavioral slope in Actor condition for M1 (M2) and Observer condition for M2 (M1).

### Neural network model for multi-agent integration task

We used recurrent neural network (RNN) models with different architectural and optimization constraints to test two hypotheses about how ACC integrates experiential and observational evidence into cumulative switch belief. One hypothesis (H1) posits that ACC receives the experienced and observed evidence through a common identity-agnostic input while another input provides information about the identity (self/other). The other hypothesis (H2) posits that ACC receives independent inputs for experienced and observed outcomes.

#### Architectural constraints.

All models have three layers, an input layer providing three distinct inputs, a hidden layer consisting of 200 recurrently connected units, and an output layer for computing the network output.

RNNs instantiating H1 receive one input conveying information about both experienced and observed outcomes and another input for identity (Actor: −1, Observer: 1). RNNs instantiating H2 receive experienced and observational outcomes through separate inputs, with only one of them active in any given trial (the other one is set to zero). These inputs project to all hidden units, are active at the onset of each trial, and are set to 0 at other timesteps. In both cases, a third input served as a go cue instructing when the RNN has to generate an output, 𝑌, which is a scalar reflecting the cumulative evidence across trials.

For all RNNs, the input carrying outcome information is a sample from a bimodal Gaussian distribution (Equation 9), with one positive and one negative mode centered at 0.5 and −0.5, corresponding to win (rewarded) and lose (unrewarded) states, respectively.

Eq. 9 $$P(x)=\frac{1}{2}\cdot N(x|\mu =-0.5,\sigma =0.1)+\frac{1}{2}\cdot N(x|\mu =0.5,\sigma =0.1)$$

For both H1 and H2, we tested two model variants. In one variant, we assumed the readout weights that drive the output were learnable (H1^learn^, H2^learn^), and in the other, the readout weights were initialized randomly and were not learnable (H1^fix^, H2^fix^). The weights of the input layer were initialized randomly and were not subjected to learning.

#### Optimization constraints.

All RNNs were trained to adjust their output, 𝑌, according to the following requirements:

- 𝑌 must reset to zero after rewarded trials (positive inputs).
- 𝑌 must integrate outcome input following unrewarded trials (negative inputs) for both Actor and Observer conditions and maintain the integrated value across trials.
- To replicate the Actor/Observer behavioral asymmetry, Actor and Observer inputs must be integrated with a gain of 1 and 0.5, respectively.
- In the first variant (H1^learn^, H2^learn^), both the recurrent and readout weights were trained. In the second variant (H1^fix^, H2^fix^), training was applied only to the recurrent weights.
- In the third variant (H1^wide^, H2^wide^), we explicitly encouraged separation between Actor and Observer outcome representations by adding a penalty term to the loss function proportional to the squared cosine similarity between the corresponding readout vectors. In principle, networks under H1 may learn effectively orthogonal representations by forming two subpopulations, with each representing Actor/Observer outcome using the identity input as a gate. In practice, however, this solution may be too fragile and difficult to reach through gradient descent.

Information was presented in a trial-based manner, with each trial having a variable duration sampled uniformly between 10 to 20 timesteps. The input was provided at the first timestep and then set to zero. A go cue input instructed the RNN when to generate an output. This go cue was presented as a linear ramp from 0 to 1 over 5 timesteps, remaining at 1 for an additional 5 timesteps. The onset of the go cue, relative to the trial start, was sampled uniformly from 0 to 5 timesteps. RNNs were required to compute and maintain the output while the go cue is 1.

#### Model dynamics.

The activity of hidden units is given by

Eq. 10 $$\tau \frac{dx}{dt}=-x(t)+W_{e}I(t)+W_{i}f(x(t))+\text{b}$$

Eq. 11 $$Y(t)=W_{o}x(t)$$

In Equation 10, τ=5, x(t) is the activity of all units, $W_{e}$ is the embedding weights for inputs, I(t), $W_{i}$ is the recurrent weights, f is a tanh nonlinear function, and b is a bias term. In Equation 11, Y(t) is the output and $W_{o}$ is the readout weights. All parameters were randomly initialized from a normal distribution with zero mean and variance 1/N, where N is the number of parameters for each layer. We trained the networks using gradient descent in batches of 16, by minimizing the MSE loss between output and target (when the go cue is at level 1). We do not constrain network dynamics outside of the reporting window.

We trained 100 models with random initiations per hypothesis, for a total of 600 models, and each model was trained for 100,000 iterations, with 500 timesteps per iteration.

#### Model performance.

We computed the performance of trained networks as the average output in the reporting period conditioned on input and trial history. 1R, 1NR, and 2NR were assigned in the same way as in behavioral trials.

#### Model analysis.

Similar to ACC, we applied TDR to activity in the hidden layer units to identify encoding dimensions for actor outcome, observer outcome, and switch evidence.

For Actor/Observer outcome (analyzed separately), we used the following regression:

Eq. 12 $$Z=\beta_{\text{input}}V_{\text{input}}+\beta_{0}$$

$V_{\text{input}}$ is an indicator variable for outcome (1: unrewarded; 0: rewarded).

For switch evidence, we used the following regression:

Eq. 13 $$Z=\beta_{\text{output}}V_{\text{output}}+\beta_{0}$$

$V_{\text{output}}$ is the output value at the time when the go cue reaches 1 for each trial.

## Supplementary Material

1

## Data Availability

The data is available on DANDI at https://dandiarchive.org/dandiset/001435.

## Code availability

The code for analysis in this manuscript is available at https://github.com/jazlab/RC_SR_NV_SBY_MJ_2025.

## References

[1] Hayden BY, Pearson JM & Platt ML Neuronal basis of sequential foraging decisions in a patchy environment. Nat Neurosci 14, 933–939 (2011). 21642973 10.1038/nn.2856 PMC3553855

[2] Shenhav A, Botvinick MM & Cohen JD The expected value of control: an integrative theory of anterior cingulate cortex function. Neuron 79, 217–240 (2013). 23889930 10.1016/j.neuron.2013.07.007 PMC3767969

[3] Sarafyazd M & Jazayeri M Hierarchical reasoning by neural circuits in the frontal cortex. Science 364, (2019). 10.1126/science.aav8911 31097640

[4] Carter C Anterior cingulate cortex, error detection, and the online monitoring of performance. Science 280, 747–749 (1998). 9563953 10.1126/science.280.5364.747

[5] Ito S, Stuphorn V, Brown JW & Schall JD Performance monitoring by the anterior cingulate cortex during saccade countermanding. Science 302, 120–122 (2003). 14526085 10.1126/science.1087847

[6] Seo H & Lee D Temporal filtering of reward signals in the dorsal anterior cingulate cortex during a mixed-strategy game. J Neurosci 27, 8366–8377 (2007). 17670983 10.1523/JNEUROSCI.2369-07.2007 PMC2413179

[7] Monosov IE Anterior cingulate is a source of valence-specific information about value and uncertainty. Nat. Commun 8, 1–12 (2017). 28747623 10.1038/s41467-017-00072-y PMC5529456

[8] Kennerley SW, Behrens TEJ & Wallis JD Double dissociation of value computations in orbitofrontal and anterior cingulate neurons. Nat Neurosci 14, 1581–1589 (2011). 22037498 10.1038/nn.2961 PMC3225689

[9] Kennerley SW & Wallis JD Evaluating choices by single neurons in the frontal lobe: outcome value encoded across multiple decision variables. Eur J Neurosci 29, 2061–2073 (2009). 19453638 10.1111/j.1460-9568.2009.06743.x PMC2715849

[10] Shima K & Tanji J Role for cingulate motor area cells in voluntary movement selection based on reward. Science 282, 1335–1338 (1998). 9812901 10.1126/science.282.5392.1335

[11] Amiez C, Joseph J-P & Procyk E Anterior cingulate error-related activity is modulated by predicted reward. Eur J Neurosci 21, 3447–3452 (2005). 16026482 10.1111/j.1460-9568.2005.04170.x PMC1913346

[12] Hadland KA, Rushworth MFS, Gaffan D & Passingham RE The anterior cingulate and reward-guided selection of actions. J. Neurophysiol 89, 1161–1164 (2003). 12574489 10.1152/jn.00634.2002

[13] Williams ZM, Bush G, Rauch SL, Cosgrove GR & Eskandar EN Human anterior cingulate neurons and the integration of monetary reward with motor responses. Nat. Neurosci 7, 1370–1375 (2004). 15558064 10.1038/nn1354

[14] Rushworth MFS & Behrens TEJ Choice, uncertainty and value in prefrontal and cingulate cortex. Nat. Neurosci 11, 389–397 (2008). 18368045 10.1038/nn2066

[15] Akam T The Anterior Cingulate Cortex Predicts Future States to Mediate Model-Based Action Selection. Neuron 109, 149–163.e7 (2021). 33152266 10.1016/j.neuron.2020.10.013 PMC7837117

[16] Vertechi P Inference-Based Decisions in a Hidden State Foraging Task: Differential Contributions of Prefrontal Cortical Areas. Neuron (2020) doi:10.1016/j.neuron.2020.01.017. PMC7146546 32048995

[17] Shidara M & Richmond BJ Anterior cingulate: single neuronal signals related to degree of reward expectancy. Science 296, 1709–1711 (2002). 12040201 10.1126/science.1069504

[18] Narayanan NS, Cavanagh JF, Frank MJ & Laubach M Common medial frontal mechanisms of adaptive control in humans and rodents. Nat. Neurosci 16, 1888–1895 (2013). 24141310 10.1038/nn.3549 PMC3840072

[19] Quilodran R, Rothé M & Procyk E Behavioral shifts and action valuation in the anterior cingulate cortex. Neuron 57, 314–325 (2008). 18215627 10.1016/j.neuron.2007.11.031

[20] Tervo DGR The anterior cingulate cortex directs exploration of alternative strategies. Neuron 109, 1876–1887.e6 (2021). 33852896 10.1016/j.neuron.2021.03.028

[21] Tervo DGR Behavioral variability through stochastic choice and its gating by anterior cingulate cortex. Cell 159, 21–32 (2014). 25259917 10.1016/j.cell.2014.08.037

[22] Bernacchia A, Seo H, Lee D & Wang X-J A reservoir of time constants for memory traces in cortical neurons. Nat. Neurosci 14, 366–372 (2011). 21317906 10.1038/nn.2752 PMC3079398

[23] Kerns JG Anterior cingulate conflict monitoring and adjustments in control. Science 303, 1023–1026 (2004). 14963333 10.1126/science.1089910

[24] Gu B-M Neural correlates of cognitive inflexibility during task-switching in obsessive-compulsive disorder. Brain 131, 155–164 (2008). 18065438 10.1093/brain/awm277

[25] Kennerley SW, Walton ME, Behrens TEJ, Buckley MJ & Rushworth MFS Optimal decision making and the anterior cingulate cortex. Nat Neurosci 9, 940–947 (2006). 16783368 10.1038/nn1724

[26] Chen W, Liang J, Wu Q & Han Y Anterior cingulate cortex provides the neural substrates for feedback-driven iteration of decision and value representation. Nat. Commun 15, 6020 (2024). 39019943 10.1038/s41467-024-50388-9 PMC11255269

[27] Joiner J, Piva M, Turrin C & Chang SWC Social learning through prediction error in the brain. NPJ Sci Learn 2, 8 (2017). 30631454 10.1038/s41539-017-0009-2 PMC6220304

[28] Grabenhorst F, Báez-Mendoza R, Genest W, Deco G & Schultz W Primate Amygdala Neurons Simulate Decision Processes of Social Partners. Cell 177, 986–998.e15 (2019). 30982599 10.1016/j.cell.2019.02.042 PMC6506276

[29] Báez-Mendoza R, Harris CJ & Schultz W Activity of striatal neurons reflects social action and own reward. Proc Natl Acad Sci U S A 110, 16634–16639 (2013). 24062436 10.1073/pnas.1211342110 PMC3799314

[30] Burke CJ, Tobler PN, Baddeley M & Schultz W Neural mechanisms of observational learning. Proc. Natl. Acad. Sci. U. S. A 107, 14431–14436 (2010). 20660717 10.1073/pnas.1003111107 PMC2922583

[31] Cooper JC, Dunne S, Furey T & O’Doherty JP Human dorsal striatum encodes prediction errors during observational learning of instrumental actions. J. Cogn. Neurosci 24, 106–118 (2012). 21812568 10.1162/jocn_a_00114 PMC3576883

[32] Azzi JCB, Sirigu A & Duhamel J-R Modulation of value representation by social context in the primate orbitofrontal cortex. Proc Natl Acad Sci U S A 109, 2126–2131 (2012). 22308343 10.1073/pnas.1111715109 PMC3277550

[33] Jeon D Observational fear learning involves affective pain system and Cav1.2 Ca2+ channels in ACC. Nat Neurosci 13, 482–488 (2010). 20190743 10.1038/nn.2504 PMC2958925

[34] Gariépy J-F Social learning in humans and other animals. Front. Neurosci 8, 58 (2014). 24765063 10.3389/fnins.2014.00058 PMC3982061

[35] de Araujo MFP Neuronal activity of the anterior cingulate cortex during an observation-based decision making task in monkeys. Behav. Brain Res 230, 48–61 (2012). 22342487 10.1016/j.bbr.2012.01.060

[36] Hill MR, Boorman ED & Fried I Observational learning computations in neurons of the human anterior cingulate cortex. Nat. Commun 7, 12722 (2016). 27598687 10.1038/ncomms12722 PMC5025858

[37] Allsop SA Corticoamygdala Transfer of Socially Derived Information Gates Observational Learning. Cell 173, 1329–1342.e18 (2018). 29731170 10.1016/j.cell.2018.04.004 PMC6345560

[38] Huang Z Ventromedial prefrontal neurons represent self-states shaped by vicarious fear in male mice. Nat. Commun 14, 3458 (2023). 37400435 10.1038/s41467-023-39081-5 PMC10318047

[39] Yoshida K, Saito N, Iriki A & Isoda M Social error monitoring in macaque frontal cortex. Nat Neurosci 15, 1307–1312 (2012). 22864610 10.1038/nn.3180

[40] Chang SWC, Gariépy J-F & Platt ML Neuronal reference frames for social decisions in primate frontal cortex. Nat. Neurosci 16, 243–250 (2013). 23263442 10.1038/nn.3287 PMC3557617

[41] Basile BM, Schafroth JL, Karaskiewicz CL, Chang SWC & Murray EA The anterior cingulate cortex is necessary for forming prosocial preferences from vicarious reinforcement in monkeys. PLoS Biol. 18, e3000677 (2020). 32530910 10.1371/journal.pbio.3000677 PMC7292358

[42] Hayden BY, Pearson JM & Platt ML Fictive reward signals in the anterior cingulate cortex. Science 324, 948–950 (2009). 19443783 10.1126/science.1168488 PMC3096846

[43] Bellebaum C, Jokisch D, Gizewski ER, Forsting M & Daum I The neural coding of expected and unexpected monetary performance outcomes: dissociations between active and observational learning. Behav Brain Res 227, 241–251 (2012). 22074898 10.1016/j.bbr.2011.10.042

[44] Morin O, Jacquet PO, Vaesen K & Acerbi A Social information use and social information waste. Philos Trans R Soc Lond B Biol Sci 376, 20200052 (2021). 33993762 10.1098/rstb.2020.0052 PMC8126467

[45] Nicolle A, Symmonds M & Dolan RJ Optimistic biases in observational learning of value. Cognition 119, 394–402 (2011). 21354558 10.1016/j.cognition.2011.02.004 PMC3081069

[46] Rigotti M The importance of mixed selectivity in complex cognitive tasks. Nature 497, 585–590 (2013). 23685452 10.1038/nature12160 PMC4412347

[47] Mante V, Sussillo D, Shenoy KV & Newsome WT Context-dependent computation by recurrent dynamics in prefrontal cortex. Nature 503, 78–84 (2013). 24201281 10.1038/nature12742 PMC4121670

[48] Ebitz RB & Hayden BY The population doctrine in cognitive neuroscience. Neuron 109, 3055–3068 (2021). 34416170 10.1016/j.neuron.2021.07.011 PMC8725976

[49] Elsayed GF, Lara AH, Kaufman MT, Churchland MM & Cunningham JP Reorganization between preparatory and movement population responses in motor cortex. Nat. Commun 7, 13239 (2016). 27807345 10.1038/ncomms13239 PMC5095296

[50] Johnston WJ, Fine JM, Yoo SBM, Ebitz RB & Hayden BY Semi-orthogonal subspaces for value mediate a binding and generalization trade-off. Nat Neurosci 27, 2218–2230 (2024). 39289564 10.1038/s41593-024-01758-5 PMC12063212

[51] Flesch T, Juechems K, Dumbalska T, Saxe A & Summerfield C Orthogonal representations for robust context-dependent task performance in brains and neural networks. Neuron 110, 4212–4219 (2022). 36549271 10.1016/j.neuron.2022.12.004 PMC9796806

[52] Tang C, Herikstad R, Parthasarathy A, Libedinsky C & Yen S-C Minimally dependent activity subspaces for working memory and motor preparation in the lateral prefrontal cortex. Elife 9, (2020). 10.7554/eLife.58154 PMC7481007 32902383

[53] Bernardi S The Geometry of Abstraction in the Hippocampus and Prefrontal Cortex. Cell 183, 954–967.e21 (2020). 33058757 10.1016/j.cell.2020.09.031 PMC8451959

[54] Remington ED, Narain D, Hosseini EA & Jazayeri M Flexible Sensorimotor Computations through Rapid Reconfiguration of Cortical Dynamics. Neuron 98, 1005–1019.e5 (2018). 29879384 10.1016/j.neuron.2018.05.020 PMC6009852

[55] Lindsey JW & Issa EB Factorized visual representations in the primate visual system and deep neural networks. Elife 13, (2024). 10.7554/eLife.91685 PMC11226229 38968311

[56] Ito T Compositional generalization through abstract representations in human and artificial neural networks. in Advances in Neural Information Processing Systems (2022).

[57] Johnston WJ & Fusi S Abstract representations emerge naturally in neural networks trained to perform multiple tasks. Nat Commun 14, 1040 (2023). 36823136 10.1038/s41467-023-36583-0 PMC9950464

[58] Daie K, Fontolan L, Druckmann S & Svoboda K Feedforward amplification in recurrent networks underlies paradoxical neural coding. bioRxiv (2023)

[59] Ganguli S One-dimensional dynamics of attention and decision making in LIP. Neuron 58, 15–25 (2008). 18400159 10.1016/j.neuron.2008.01.038 PMC7204626

[60] Brown JW & Alexander WH Foraging Value, Risk Avoidance, and Multiple Control Signals: How the Anterior Cingulate Cortex Controls Value-based Decision-making. J Cogn Neurosci 29, 1656–1673 (2017). 28430040 10.1162/jocn_a_01140

[61] Vassena E, Holroyd CB & Alexander WH Computational Models of Anterior Cingulate Cortex: At the Crossroads between Prediction and Effort. Front Neurosci 11, 316 (2017). 28634438 10.3389/fnins.2017.00316 PMC5459890

[62] Mardinly AR Precise multimodal optical control of neural ensemble activity. Nat. Neurosci 21, 881–893 (2018). 29713079 10.1038/s41593-018-0139-8 PMC5970968

[63] Clark AM An optrode array for spatiotemporally-precise large-scale optogenetic stimulation of deep cortical layers in non-human primates. Commun. Biol 7, 329 (2024). 38485764 10.1038/s42003-024-05984-2 PMC10940688

[64] Russell LE All-optical interrogation of neural circuits in behaving mice. Nat. Protoc 17, 1579–1620 (2022). 35478249 10.1038/s41596-022-00691-w PMC7616378

[65] Watters N, Tenenbaum J & Jazayeri M Modular Object-Oriented Games: A Task Framework for Reinforcement Learning, Psychology, and Neuroscience. (2021).

